# Walking pace and its association with osteoporosis and pathological fractures: insights from UK biobank

**DOI:** 10.3389/fendo.2025.1635999

**Published:** 2025-08-26

**Authors:** Qin Hu, Haoran Liu, Yuchen Du, Runchen Duan, Linpeng Li, Daishui Yang, Zhengxiao Ouyang

**Affiliations:** ^1^ Department of Orthopedics, The Second Xiangya Hospital, Central South University, Changsha, Hunan, China; ^2^ Emergency Medicine Center, Sichuan Provincial People’s Hospital, University of Electronic Science and Technology of China, Chengdu, China

**Keywords:** osteoporosis, walking pace, bone mineral density, physical activity, UK Biobank

## Abstract

**Backgrounds:**

Osteoporosis is a prevalent bone disease. Previous research has indicated that various forms of exercise have different protective effects on bone health, yet there are limited studies on the impact of walking pace on bone mineral density (BMD) and skeletal health. This study aims to investigate the correlation between usual walking pace and osteoporosis, including related fractures, while considering potential interactions with genetic vulnerability.

**Methods:**

Survey responses from 348,334 participants in the UK Biobank were analyzed, obtaining information on usual walking pace, BMD, osteoporosis incidence, and genetic vulnerability. Multiple linear regression, Cox proportional hazards regression models, and stratified analysis methods were employed. A weighted genetic risk score for osteoporosis was calculated.

**Results:**

Cross-sectional analysis revealed a notable upward trend in BMD and a downward trend in the risk of osteoporosis and fractures from slow to brisk walking pace (*P* <0.001). Slow walking was associated with the highest risk of osteoporosis [hazard ratio (HR) 2.18, 95% confidence interval (CI) 2.03 - 2.34] and fractures (HR 2.25, 95% CI 1.79 - 2.81). Prospective analysis showed that brisk walking was significantly linked to reduced incident osteoporosis (HR 0.85, 95% CI 0.79 - 0.91) and fractures (HR 0.75, 95% CI 0.63 - 0.89) after excluding baseline cases.

**Conclusions:**

The findings provide evidence that walking pace is closely related to the prevalence of osteoporosis and fracture incidence. Therefore, integrating walking into national physical activity initiatives and encouraging individuals to increase their walking pace could serve as an effective preventive measure against osteoporosis.

## 1 Introduction

Osteoporosis is a common bone condition characterized by decreased bone mineral density (BMD), microarchitectural deterioration, and an increased susceptibility to fractures (1, 2). Alongside reduced BMD and diminished connectivity (3, 4), Osteoporosis poses a significant health risk (5). According to data from the International Osteoporosis Foundation, in 2017, a proximately 2.8 million individuals aged 50 and above in the UK suffered from osteoporosis, with medical expenses for treating this condition soaring to £4.5 billion. Furthermore, projections suggest that this figure could reach £5.9 billion by the year 2030 (6). Today, osteoporosis has evolved into a significant public health concern worldwide (7). Therefore, given the severity of the consequences of osteoporosis and the improvement in human living standards, it is essential to make necessary enhancements in prevention strategies for osteoporosis.

While numerous studies have validated the importance of diminishing sedentary habits and amplifying physical activity to uphold BMD and diminish osteoporosis rates (8, 9), ongoing research predominantly delves into diverse intensities and forms of physical exercises, including resistance training and high-intensity activities (10, 11), tailored to distinct demographic groups (like swimming (12), running and jumping (13)). Moreover, research indicates that different walking paces are closely related to various common diseases and can predict the future prevalence or prognosis of certain diseases within a population. For example, studies shows that a brisk walking pace can reduce the risk of chronic diseases such as type 2 diabetes (14), heart failure (15), chronic obstructive pulmonary disease (COPD) (16), and Alzheimer’s disease (17), while a slow walking pace is associated with a higher risk of these diseases. Additionally, studies have shown that the rehabilitation strategy of brisk walking can improve the cognitive ability of Parkinson’s patients (18). Some studies have also found that brisk walking may be associated with longer leukocyte telomere length (19). A 2019 cohort study published in JAMA found that a slow walking pace is associated with a faster aging process and an older facial appearance (20). These findings suggest a significant and potentially exploitable relationship between walking pace and various aging-related diseases. However, research on the link between walking pace and osteoporosis, a common age-related condition, remains limited, indicating valuable research potential. As a result, we embarked upon this research endeavor.

Utilizing data from 348,334 UK Biobank participants, our study explored the link between walking pace, BMD, and osteoporosis. We investigated the correlation between walking pace and genetic susceptibility to osteoporosis, and further examined this relationship after excluding baseline osteoporosis. These insights inform targeted health recommendations and policies for osteoporosis prevention.

## 2 Materials and methods

### 2.1 Study design and participants

**Figure 1:**
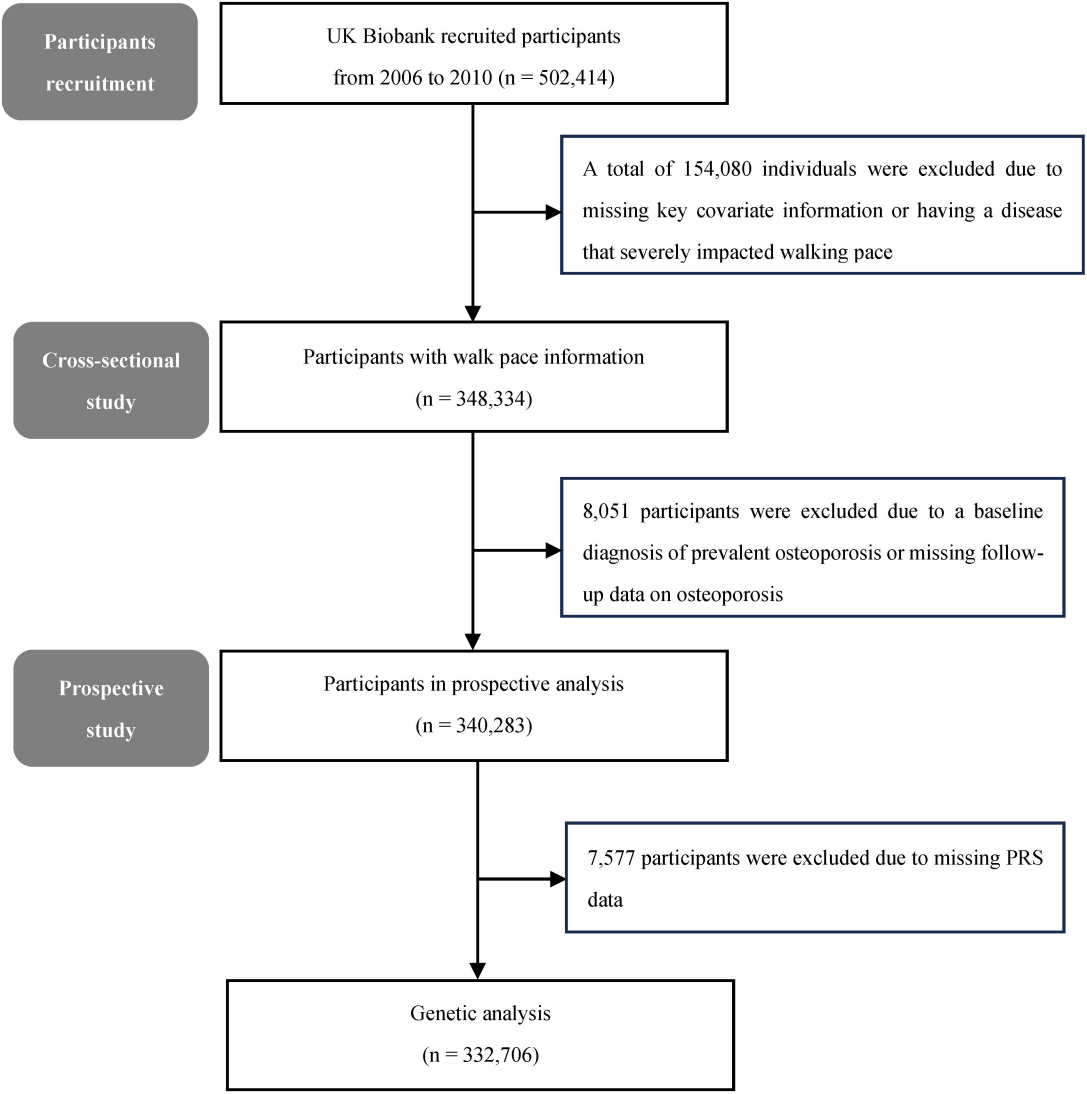
Flowchart of selection process.

A prospective cohort study based on a population of 500,000 people was conducted in the United Kingdom from 2006 to 2010, which participants were predominantly 40–69 years old. All participants signed an informed consent form and ethics approval was obtained from the North West Multicenter Research Ethics Committee. During recruitment, participants were asked about their medical conditions. A total of 154,080 individuals were excluded due to missing key covariate information (e.g., walking pace, osteoporosis) or a medical history that could significantly impact walking pace, such as heart attack, angina, stroke, asthma, deep vein thrombosis, pulmonary embolism, emphysema, cancer, or fractures within the past five years. 348,334 participants met the inclusion criteria and were available for analysis. The flowchart is shown in **Figure 1**.

### 2.2 Walking pace assessment

We used the average walking pace per hour (Field ID: 924) to define usual walking pace. A slow walking pace is characterized as being under 3 miles per hour. An average pace is considered to be between 3 and 4 miles per hour, while a brisk pace is identified as exceeding 4 miles per hour.

### 2.3 Osteoporosis evaluation

Estimated bone mineral density (eBMD) was determined using the Sahara heel ultrasound system (Hologic, USA). To calculate BMD (g/cm^2^), we combined the speed of sound (SOS, in m/s) and bone ultrasound attenuation (BUA, in dB/MHz) using the formula eBMD = (SOS + BUA) * 0.002592 − 3.687 (21). Additionally, T-scores were used to express the deviation of an individual’s BMD from that of healthy adults of the same gender, indicating the number of standard deviation (SD) between the measured value and the norm. Osteoporosis was categorized as prevalent with a T-score of ≤ −2.5 (22). Detailed procedures and quality control measures can be found at https://biobank.ndph.ox.ac.uk/showcase/refer.cgi?id=100248.

While heel eBMD has shown moderate correlation with dual-energy X-ray absorptiometry (DXA)-based measurements and is widely used in large-scale cohorts, it may not fully capture axial skeletal density (e.g., spine and hip), which is more predictive of fracture risk. Additionally, quantitative heel ultrasound (QUS)-based estimates may be less precise in classifying osteoporosis status.

Osteoporosis cases that occurred during the follow-up period were verified through self-reported data and medical records, including hospital admissions, primary care notes, and death registry data. This approach aligns with the methodology used in earlier UK Biobank research (23, 24). Diagnoses were obtained via self-reporting during verbal interviews. Hospital inpatient data were linked to Health Episode Statistics and the Scottish Morbidity Records. Primary care data were gathered from various general practice records across the UK. Death registration information was sourced from the National Health Service Information Center and the NHS Central Register Scotland. Osteoporosis was diagnosed based on the criteria set by the International Classification of Diseases, Tenth Revision (codes M80 − M82). To improve diagnostic precision for new cases of osteoporosis, individuals already diagnosed with osteoporosis at baseline (n = 11,875) were excluded from the study. Participants were monitored until an osteoporosis event, death, or until 31 October 2022, whichever came first.

### 2.4 Genetic risk score for osteoporosis

Individuals’ vulnerability to osteoporosis was evaluated using the standard osteoporosis PRS (Field ID: 26258), calculated as the sum of risk alleles weighted accordingly. The approach involved obtaining the score through Bayesian analysis, incorporating meta-analyses of summary statistics from external studies on weighted genetic scores (25). The calculation process for osteoporosis PRS is accessible through https://biobank.ndph.ox.ac.uk/showcase/refer.cgi?id=5202. Participants were categorized into three groups based on their genetic risk of osteoporosis: high (top tertile: 66.7% and above), moderate (middle tertile: between 33.3% and 66.7%), and low (bottom tertile: below 33.3%).

### 2.5 Covariates

The current study encompassed certain demographic details and osteoporosis-related health metrics endorsed by the American Academy of Orthopedic Surgery (AAOS), including demographic, socioeconomic, lifestyle, and genetic factors. The general information encompassed age (40 - 69), sex (male/female), body mass index (BMI, kg/m^2^), education (college or university/others), Townsend index, and ethnicity (White/others). According to the World Health Organization’s classification, BMI was divided into four categories: underweight (<18.5 kg/m²), normal weight (18.5–24.9 kg/m²), overweight (25.0–29.9 kg/m²), and obese (≥30.0 kg/m²). The potential confounders primarily encompassed: smoking status (current/never or former), alcohol consumption (current/never or former), metabolic equivalent task minutes per week (an equivalent combination of ≥ 75 in vigorous intensity, ≥ 150 in moderate intensity, and a missing indicator category), dietary habits (comprising vegetable, fruit, fish, unprocessed and processed meat intake) (26).

### 2.6 Statistical analysis

Statistical analyses were conducted using IBM SPSS Statistics, version 27 (IBM Corporation, Armonk, NY, USA), along with the integrated R package (27). Continuous variables were presented as mean ± SD, and categorical variables were depicted as percentages. Statistical significance was inferred at a significance level of *p*<0.05. The baseline characteristics of the study population are reported as the means or percentages by walk pace as “normal pace, slow pace or brisk pace”. The duration of follow-up was determined as the period from the recruitment date to the occurrence of osteoporosis diagnosis, death, or the censoring date (31 October 2022), whichever event transpired first.

Initially, we utilized a linear regression model to investigate the correlation between usual walking pace and BMD. To examine the relationship between walking pace and prevalent osteoporosis, a logistic regression model was applied. Additionally, Cox proportional hazard models were used to determine the hazard ratios (HRs) and the 95% confidence intervals (95% CI) for the link between walking pace and the occurrence of osteoporosis and fractures. We adjusted for several confounders, including age, gender, BMI, ethnicity, education level, Townsend deprivation index, alcohol consumption, smoking status, physical activity, and healthy diet score.

Moreover, to evaluate the consistency of the association between walking pace and the risk of incident osteoporosis, we conducted stratified analyses. These analyses were based on variables including sex, BMI, smoking status, alcohol consumption, and physical activity status. The assessment was carried out using a log-likelihood ratio test by comparing models with and without cross-product interaction terms.

Our investigation delved deeper into whether the connection between the usual walking pace and osteoporosis risk could be impacted by genetic predisposition to osteoporosis. In the interaction analysis, P-values for interaction were calculated using a log-likelihood ratio test to assess the differences between models that included cross-product interaction terms and those that did not. Utilizing a multivariable-adjusted regression model, we investigated the interaction between the tertile-categorized PRS with osteoporosis. Subsequently, a stratified analysis was conducted based on PRS categories and usual walking pace (28).

In the sensitivity analyses, we excluded participants with incident osteoporosis ≥ 2 years and those with osteopenia (T-score < −1) from the baseline to perform the regression.

## 3 Results

### 3.1 Sample characteristics

In accordance with usual walking pace status, **Table 1** displays the baseline characteristics of the study population. Within the cohort of 348,334 participants, the distribution of ‘slow pace,’ ‘normal pace,’ and ‘brisk pace’ accounted for 6.5%, 52.8%, and 40.7% of the population, respectively. Compared to the ‘normal pace’ group, individuals in the ‘slow pace’ category tended to be female, relatively older, with lower educational attainment, and a lower prevalence of socio-economic disadvantages. Additionally, this group exhibited a higher representation of non-European ethnicities, higher BMI, lower levels of physical activity, smoking rates and alcohol consumption. However, in the ‘brisk pace’ group, the baseline characteristics of the study population were exactly opposite to those of the ‘slow pace’ group.

**Table 1:** Demographic characteristics of participants by walk pace in the UK Biobank cohorts at the baseline.

Variables	Walk pace		
	Slow pace	Normal pace	Brisk pace
No.of participants (%)	22,717 (6.5)	183,927 (52.8)	141,690 (40.7)
Age (median & range, years)	60.0 (53.0 - 64.0)	58.0 (50.0 - 63.0)	55.0 (48.0 - 62.0)
Sex (women, %)	13,292 (58.5)	100,711 (54.8)	76,334 (53.9)
BMI (median & range, kg/m^2^)	30.4 (26.6 - 34.9)	27.3 (24.7 - 30.5)	25.4 (23.2 - 28.0)
Education (college or university, %)	4,734 (21.3)	53,753 (29.6)	115,533 (33.5)
Ethnic (White, %)	19,788 (87.7)	171,304 (93.4)	327,288 (94.3)
Townsend deprivation index	-0.9 (-3.0 - 2.4)	-2.2 (-3.7 - 0.4)	-2.4 (-3.8 - -0.1)
Smoke (current, %)	3,616 (16.0)	19,250 (10.5)	11,832 (8.4)
Drinks (current, %)	18,577 (82.1)	169,459 (92.2)	133,725 (94.4)
Physical activity			
At goal	9,292 (40.9)	116,150 (63.2)	103,797 (73.3)
Not at goal	7,019 (30.9)	30,103 (16.4)	17,301 (12.2)
Missing	6,406 (28.2)	37,674 (20.5)	20,592 (14.5)
Healthy diet (median & range)			
Vegetable (tablespoons per day)	4.0 (3.0 - 6.0)	4.0 (3.0 - 6.0)	4.0 (3.0 - 6.0)
Fruit (pieces per day)	2.0 (1.0 - 4.0)	2.0 (1.0 - 4.0)	3.0 (2.0 - 4.0)
Fish (times per week)	1.5 (1.0 - 3.0)	1.5 (1.0 - 3.5)	2.0 (1.5 - 3.5)
Unprocessed meat (times per week)	1.0 (0.5 - 3.0)	1.0 (0.5 - 3.0)	1.0 (0.5 - 3.0)
Processed meat (times per week)	4.0 (2.5 - 5.0)	4.0 (2.5 - 5.0)	4.0 (2.5 - 5.0)

BMI, body mass index.

### 3.2 Relationship between walking pace and osteoporosis, BMD and fracture

Subsequently, the correlation between the usual walking pace and BMD (g/cm^2^), as well as the prevalence of osteoporosis at the initial assessment, was analyzed (**Table 2**). The outcomes from the multiple linear regression analysis revealed that in the ultimate model following variable adjustments, based on normal walking pace, reduced walking pace correlated with decreased BMD (β = −0.017, 95% CI: −0.020 to −0.015), whereas brisk walking pace correlated with elevated BMD (β = 0.003, 95% CI 0.002 to 0.004). The results from the binary logistic regression analysis, adjusting for multiple variables, demonstrated a significant correlation between slow walking pace and prevalent osteoporosis when compared to normal pace (OR = 2.32, 95% CI: 2.09 to 2.57). Conversely, brisk walking pace was linked to a reduced likelihood of osteoporosis.

**Table 2:** Association between current walk pace and bone mineral density (g/cm^2^) and prevalent osteoporosis at the baseline.

Bone mineral density	Multiple linear regression [β (95%CI), *p* - value]				
	Normal pace	Slow pace		Brisk pace	
Model 1	1.00 (reference)	-0.018 (-0.020 - -0.016)	<0.001	0.004 (0.003 - 0.005)	<0.001
Model 2	1.00 (reference)	-0.021 (-0.023 - -0.019)	<0.001	0.005 (0.004 - 0.006)	<0.001
Model 3	1.00 (reference)	-0.017 (-0.020 - -0.015)	<0.001	0.003 (0.002 - 0.004)	<0.001
Prevalent osteoporosis	Binary logistic regression [OR (95%CI), *p* - value]				
	Normal pace	Slow pace		Brisk pace	
Model 1	1.00 (reference)	2.64 (2.41 - 2.90)	<0.001	0.77 (0.72 - 0.83)	<0.001
Model 2	1.00 (reference)	2.42 (2.20 - 2.66)	<0.001	0.81 (0.75 - 0.87)	<0.001
Model 3	1.00 (reference)	2.32 (2.09 - 2.57)	<0.001	0.85 (0.78 - 0.91)	<0.001

CI, Confidence Interval; OR, Odds Ratio.

Model 1 adjusted for age, sex (male/female) and BMI.

Model 2 additional adjusted for ethnic (White/other), education (college or university/other) and Townsend index.

Model 3 additional adjusted for smoke (yes/no), drinks (yes/no), physical activity (at goal/not at goal) and healthy diet.

Following this, Cox regression analyses were conducted to investigate the correlation between the usual walking pace and the incident of osteoporosis and fractures over the entire follow-up duration (**Table 3**). In Cox proportional-hazards Model 1, adjusting for age, sex, and BMI, a notable correlation was observed between slower walking pace and a heightened risk of developing both osteoporosis and fractures (HR = 2.36, 95% CI: 2.20 to 2.52 and HR = 2.51, 95% CI: 2.03 to 3.10), while brisk walking pace was consistently associated with a lower risk of osteoporosis and fracture (HR = 0.86, 95% CI: 0.82 to 0.90 and HR = 0.75, 95% CI: 0.63 to 0.89, respectively; *P* < 0.001). Subsequently, the analysis of the multivariable adjusted models in both Model 2 and Model 3 confirmed the aforementioned association (both *P* for trend < 0.001), although the magnitude of the relationship between incident osteoporosis and fracture and slow walking pace was marginally reduced.

**Table 3:** Cox regression analysis of the association between current walking pace and the incident of osteoporosis and fractures during the follow-up.

Incident osteoporosis	Cox regression [HR (95%CI), *p* - value]				
	Normal pace	Slow pace		Brisk pace	
Model 1	1.00 (reference)	2.36 (2.21 - 2.52)	<0.001	0.86 (0.82 - 0.90)	<0.001
Model 2	1.00 (reference)	2.30 (2.15 - 2.46)	<0.001	0.86 (0.82 - 0.90)	<0.001
Model 3	1.00 (reference)	2.18 (2.03 - 2.34)	<0.001	0.87 (0.83 - 0.91)	<0.001
Incident fracture	Cox regression [HR (95%CI), *p* - value]				
	Normal pace	Slow pace		Brisk pace	
Model 1	1.00 (reference)	2.57 (2.09 - 3.17)	<0.001	0.76 (0.64 - 0.89)	0.001
Model 2	1.00 (reference)	2.49 (2.02 - 3.08)	<0.001	0.76 (0.65 - 0.90)	0.001
Model 3	1.00 (reference)	2.25 (1.79 - 2.81)	<0.001	0.78 (0.65 - 0.92)	0.004

HR, Hazard Ratio, CI, Confidence Interval.

Model 1 adjusted for age, sex (male/female) and BMI.

Model 2 additional adjusted for ethnic (White/other), education (college or university/other) and Townsend index.

Model 3 additional adjusted for smoke (yes/no), drinks (yes/no), physical activity (at goal/not at goal) and healthy diet.

When further excluding individuals with a baseline history of osteoporosis ≥ 2 years or a T score < −1, the positive association between walking pace and osteoporosis risk was not substantially modified (Supplementary file, **Supplementary Tables S1, S2**). Compared to a usual walking pace, a slow walking pace was significantly negative associated with the occurrence of osteoporosis and fractures (HR = 2.03, 95% CI: 1.82 to 2.26). Moreover, the relationship between brisk walking pace and osteoporosis and fractures, although attenuated relative to the findings in **Table 2**, still persisted (HR = 0.75, 95% CI: 0.63 to 0.89).

### 3.3 Stratified analysis of walking pace and related factors of osteoporosis

In further stratified analyses by sex, BMI, smoking status, alcohol consumption, and physical activity, we evaluated whether the association between walking pace and osteoporosis risk varied across subgroups (**Table 4**). A significant interaction was observed for sex (*p* for interaction < 0.001). Specifically, the relationship between walking pace and osteoporosis appeared stronger in males than in females. As shown in **Table 4**, a significant interaction between BMI and walking pace was observed (*p* for interaction = 0.018). The harmful effect of slow walking was more pronounced among participants with normal or underweight BMI, whereas this association was attenuated to some extent in the overweight and obese subgroups. In contrast, no significant interaction was observed for smoking, alcohol consumption, or physical activity, indicating that the relationship between walking pace and osteoporosis risk remained consistent across these factors.

**Table 4:** Stratified analysis the association of current walk pace and osteoporosis by sex, BMI, smoke, drinks and physical activity status.

Variables	Walk pace			*p* for interaction
	Normal pace	Slow pace	Brisk pace	
Sex				
Women	1.00 (reference)	2.03 (1.87 - 2.20)	0.90 (0.85 - 0.95)	<0.001
Men	1.00 (reference)	2.85 (2.44 - 3.33)	0.73 (0.65 - 0.83)
BMI (kg/m^2^)				
underweight	1.00 (reference)	2.28 (1.19 - 4.34)	0.80 (0.55 - 1.15)	0.018
normal	1.00 (reference)	2.30 (2.00 - 2.63)	0.91 (0.85 - 0.97)
overweight	1.00 (reference)	1.96 (1.74 - 2.21)	0.84 (0.77 - 0.91)
obese	1.00 (reference)	2.00 (1.77 - 2.25)	1.05 (0.90 - 1.21)
Smoke				
No	1.00 (reference)	2.23 (2.07 - 2.41)	0.88 (0.84 - 0.93)	0.074
Yes	1.00 (reference)	1.89 (1.57 - 2.29)	0.75 (0.64 - 0.89)
Drinks				
No	1.00 (reference)	2.04 (1.72 - 2.42)	0.87 (0.75 - 1.02)	0.743
Yes	1.00 (reference)	2.21 (2.04 - 2.39)	0.87 (0.83 - 0.91)
Physical activity				
At goal	1.00 (reference)	2.16 (1.95 - 2.41)	0.88 (0.83 - 0.93)	0.777
Not at goal	1.00 (reference)	2.14 (1.86 - 2.46)	0.82 (0.72 - 0.94)

HR, Hazard Ratio, CI, Confidence Interval.

*BMI stratification based on standard clinical categories: underweight (<18.5 kg/m²), normal weight (18.5–24.9 kg/m²), overweight (25.0–29.9 kg/m²), and obese (≥30.0 kg/m²).

Adjusted for age, sex, BMI, ethnic, education, Townsend index, smoke, drinks, physical activity and healthy diet.

### 3.4 Association between the effect of walking pace on osteoporosis and genetic susceptibility

The results of the Cox proportional hazards analysis (**Table 5**) indicate that there was a significant interaction between walking pace and genetic susceptibility in relation to the risk of osteoporosis (*p* for interaction = 0.003). In the low genetic predisposition group, compared with a normal walking pace, a slow or brisk walking pace was significantly associated with a higher (HR = 2.23, 95% CI: 1.93–2.59) or lower (HR = 0.81, 95% CI: 0.73–0.90) risk of osteoporosis, respectively. In contrast, in the high genetic predisposition group, slow and brisk walking paces were associated with less pronounced effects on osteoporosis risk (HR = 2.03, 95% CI: 1.81–2.27; and HR = 0.90, 95% CI: 0.83–0.96, respectively).

**Table 5:** Association of current walk pace and osteoporosis incident hazard ratio by genetic predisposition.

Group	HR (95% CI)	*p* for interaction
Low genetic group		0.003
Normal pace	1.00 (reference)	
Slow pace	2.23 (1.93 - 2.59)	
Brisk pace	0.81 (0.73 - 0.90)	
Medium genetic group		
Normal pace	1.00 (reference)	
Slow pace	2.27 (2.00 - 2.56)	
Brisk pace	0.88 (0.81 - 0.96)	
High genetic group		
Normal pace	1.00 (reference)	
Slow pace	2.03 (1.81 - 2.27)	
Brisk pace	0.90 (0.83 - 0.96)	

HR, Hazard Ratio, CI, Confidence Interval.

Adjusted for age, sex, BMI, ethnic, education, Townsend index, smoke, drinks, physical activity and healthy diet.

## 4 Discussion

In this large-scale prospective cohort study, the study has made the following discoveries: 1) in the multivariable-adjusted model, slow walking pace is associated with lower BMD and a higher risk of osteoporosis, whereas brisk walking pace is associated with higher BMD and a lower risk of osteoporosis. 2) compared to normal walking pace, slow walking not only increases the incidence of osteoporosis but also raises the risk of incident fracture, whereas brisk walking pace reduces the risk of both. 3) gender-stratified analysis results indicate that slow walking pace increases the incidence of osteoporosis in both men and women, with a slightly greater impact observed in men compared to women. 4) the relationship between walking pace and osteoporosis risk is also influenced by BMI but is unrelated to smoking, alcohol consumption, and physical activity. 5) the inverse correlation between usual walking pace and osteoporosis persisted irrespective of low, moderate, or high genetic risk.

Previous research has predominantly focused on the elderly or regarded walking pace as one of the predictive factors. Findings from a decade-long prospective investigation conducted in 2014 to anticipate fracture vulnerability in elderly women revealed a distinct correlation: diminished walking pace emerged as a stand-alone predictor linked to heightened susceptibility to fractures, and correlates with osteoporosis-related fractures (29). In a recent investigation, parallel conclusions were drawn: a noteworthy link was established between diminished walking pace and a heightened likelihood of hip fractures among aging women (30). Our study, primarily focused on investigating the correlation between walking pace and the risk of osteoporosis and fractures, transcends the confines of the elderly population, unveiling a distinct relationship between the two.

The current findings of our study indicate: a slow walking pace markedly elevated the incidence of osteoporosis and the risk of fractures in comparison to normal walking pace, whereas brisk walking potentially mitigated both risks. In their research investigating the predictive value of gait dynamics parameters on femur BMD in individuals over 50 years old, Wooyoung Choi et al. (31). discovered that walking plays a pivotal role in preserving BMD among older adults, with walking exercises demonstrating a potential decrease in osteoporosis prevalence. This aligns closely with the findings of our study.

Numerous investigations have documented the connection between BMD and diverse forms of physical activities, including impact loading exercises (32, 33) (such as jumping and weight-bearing exercises) as well as resistance training (13, 34). Based on these findings, there is a suggestion that physical activity may have an impact on BMD and the occurrence of osteoporosis to a certain degree; nonetheless, only a limited number of studies have delved into the connection between BMD, osteoporosis, and walking pace. Hence, this study aims to fill this particular void by delving deeply into the correlation between walking pace and osteoporosis.

The interaction between gender and walking pace shows difference in its impact on osteoporosis. Both genders exhibit a significant correlation, with males showing a slightly higher risk of osteoporosis with slow walking compared to females. The difference may stem from men generally having greater muscle mass, as bone density is largely influenced by muscle load and tension. When walking speed slows, reduced muscle activity lessens mechanical stimulation of bones, accelerating bone loss. In contrast, faster gait enhances muscle activity, providing stronger bone protection (35, 36). Lifestyle factors may also play a role: men often overlook minor falls or early bone issues, showing significant risk only when gait changes markedly, while women tend to seek medical care earlier, which may weaken the predictive value of gait changes for osteoporosis risk in women (37). Nonetheless, certain prior reports in this field have showcased a more pronounced impact of exercise on BMD in women (32), while in some other studies, conflicting results have been reported in men (38). The gender-based variations observed could potentially be attributed to differences in sample sizes, warranting further in-depth investigation in subsequent studies.

Our stratified analysis results further reveal a significant interaction effect between BMI and walking pace on osteoporosis. The results indicate that the interaction between BMI and walking pace has a significant effect on osteoporosis. Our stratified analysis suggests that the association between walking pace and osteoporosis may be modified by BMI. The inverse relationship between brisk walking pace and osteoporosis was most evident among individuals with normal BMI, while the association was weaker and not statistically significant in underweight or obese participants. This indicates that body composition may influence how walking pace relates to bone health. Conversely, the interaction of smoking, alcohol consumption, and physical activity with walking pace did not significantly affect the incidence of osteoporosis. Further investigation is warranted to thoroughly explore the interplay between these factors and walking pace to osteoporosis.

There are several plausible biological mechanisms underlying the positive association between walking pace and BMD, as well as the inverse association with osteoporosis and incident fractures. Walking pace is widely recognized not only as a general indicator of overall health but also as a key marker of functional capacity and physiological reserve (39). Individuals with faster walking speeds tend to exhibit better overall health and greater ability to engage in moderate-to-vigorous physical activities, which may lead to enhanced mechanical loading on bones and improved physical fitness (40). Secondly, brisk walking has been shown to benefit cardiopulmonary function (41, 42), which may in turn improve systemic circulation and reduce the risk of osteoporosis. Improved vascular health could promote bone remodeling and enhance bone quality through increased nutrient and oxygen delivery (43, 44), ultimately leading to higher bone density and reduced fracture risk. Thirdly, muscle strength—a well-established marker of skeletal health—is strongly associated with whole-body BMD (45). Walking pace has been positively correlated with muscle mass (46), while systemic inflammation has been linked to sarcopenia and bone loss (47, 48). Therefore, a faster walking pace may help preserve or increase muscle mass and bone density, potentially by attenuating systemic inflammatory levels, thereby reducing the risk of osteoporosis and related fractures.

Additionally, we also investigate the relationship between walk pace and polygenetic risk score for osteoporosis. The results underscore that irrespective of an individual’s genetic predisposition level (low, intermediate, or high), the relationship between walking pace and the risk of osteoporosis remains steadfast, with a notable interaction noted: slow walking pace escalates the incidence of osteoporosis, whereas brisk walking pace diminishes the risk of developing osteoporosis. Therefore, fundamentally speaking, slow walking appears to be an independent risk factor for osteoporosis, unrelated to genetic predisposition. However, it is noteworthy that this study primarily focused on the White British population (>90%), lacking investigation into other ethnic groups. To gain a more comprehensive understanding of the relationship between these factors, further exploration into the genetic susceptibility of diverse populations is warranted. These preliminary research findings suggest that regardless of genetic susceptibility, increasing walking pace may be beneficial in reducing the risk of developing osteoporosis.

Our study has several limitations. First, as an observational study, it identifies associations between walking pace and osteoporosis but cannot establish causality. Second, walking pace was self-reported, which may introduce recall bias and measurement variability. Although practical in large cohorts, this method may not capture actual gait performance. Future studies should use objective tools (e.g., accelerometers) for validation. Third, we excluded cases with osteoporosis or fractures diagnosed ≥2 years prior to baseline, but undetected cases within that window may still influence results. Fourth, information on fracture sites was not systematically recorded or followed up in the UK Biobank, limiting our ability to assess site-specific associations with walking pace. Fifth, since walking pace was collected as a categorical variable, we were unable to apply methods such as restricted cubic splines to assess potential non-linear associations. Sixth, despite adjusting for key confounders, residual confounding from unmeasured variables cannot be ruled out. Seventh, the UK Biobank is subject to healthy volunteer bias, limiting representativeness. Finally, as most participants were White British, generalizability to other ethnic groups is limited; replication in more diverse populations is needed.

## 5 Conclusion

In summary, regardless of low, medium, or high genetic risk, walking pace is negatively correlated with the incidence of osteoporosis. Additionally, walking pace is positively correlated with BMD and negatively correlated with incidental fractures. Therefore, this study suggests that enhancing physical activity levels, improving overall fitness, and increasing walking pace could emerge as crucial strategies for osteoporosis prevention.

## Data Availability

The datasets presented in this study can be found in online repositories. The names of the repository/repositories and accession number(s) can be found below: https://www.ukbiobank.ac.uk/.
