# Supplementary figures and images for: Walking pace and its association with osteoporosis and pathological fractures: insights from UK biobank
Source: Front Endocrinol (Lausanne). 2025 Aug 26;16:1635999. doi: 10.3389/fendo.2025.1635999 (PMC12417151; doi:10.3389/fendo.2025.1635999)

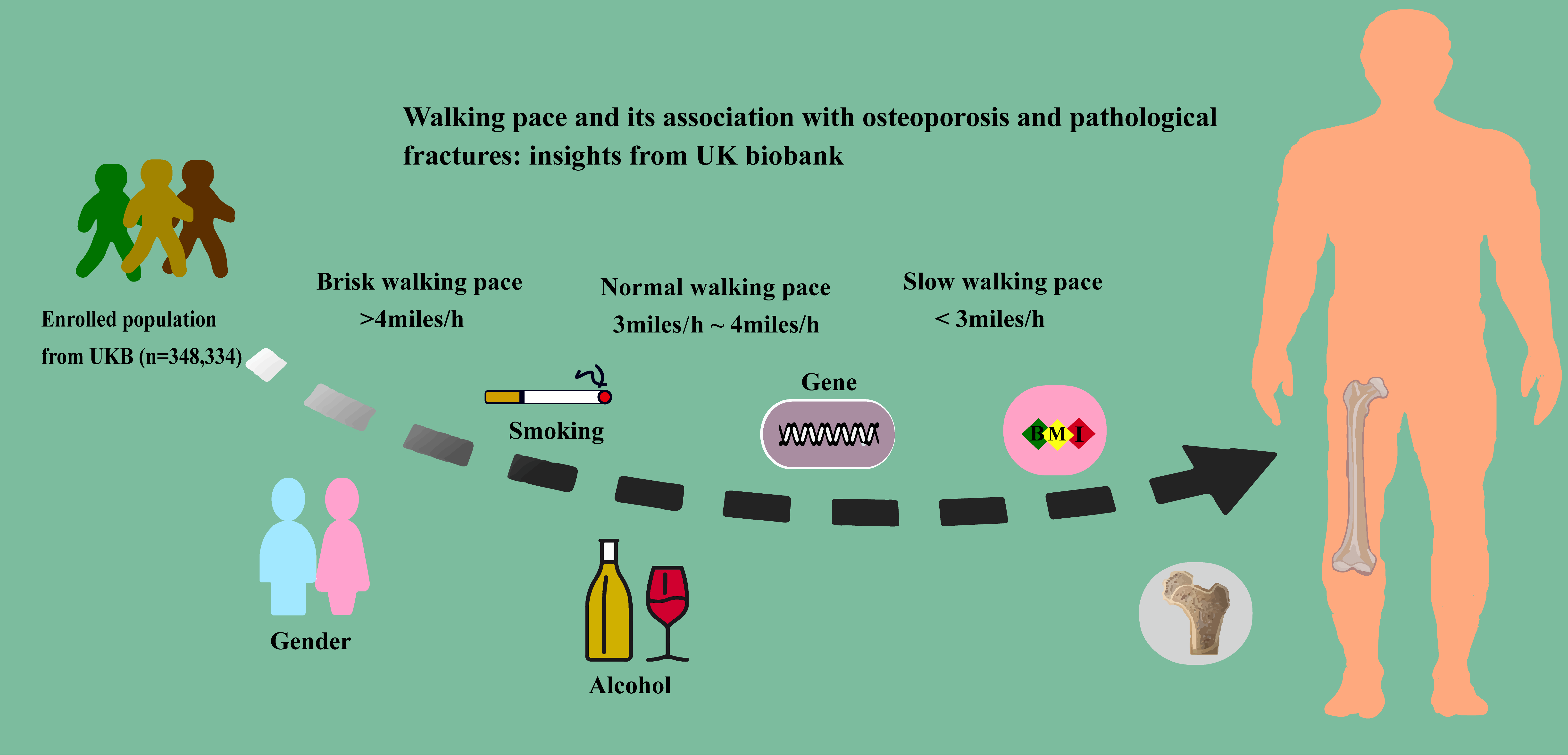

Supplement: Supplementary file 1 [file Image1.tif]
