# Supplementary material for: Walking pace and its association with osteoporosis and pathological fractures: insights from UK biobank
Source: Front Endocrinol (Lausanne). 2025 Aug 26;16:1635999. doi: 10.3389/fendo.2025.1635999 (PMC12417151; doi:10.3389/fendo.2025.1635999)
Supplement: Supplementary file 2 [file Table1.docx]

**Supplementary Materials**

**Table S1**. Excluding participants with incident osteoporosis ≥ 2 years from the baseline.

| Incident osteoporosis | Cox regression [HR (95%CI), *p* - value] | | | | |
| --- | --- | --- | --- | --- | --- |
|  | Normal pace | Slow pace | | Brisk pace | |
| Model 1 | 1.00 (reference) | 2.36 (2.20 - 2.52) | <0.001 | 0.86 (0.82 - 0.90) | <0.001 |
| Model 2 | 1.00 (reference) | 2.29 (2.14 - 2.46) | <0.001 | 0.86 (0.82 - 0.90) | <0.001 |
| Model 3 | 1.00 (reference) | 2.17 (2.02 - 2.34) | <0.001 | 0.87 (0.83 - 0.92) | <0.001 |
| Incident fracture | Cox regression [HR (95%CI), *p* - value] | | | | |
|  | Normal pace | Slow pace | | Brisk pace | |
| Model 1 | 1.00 (reference) | 2.51 (2.03 - 3.10) | <0.001 | 0.75 (0.63 - 0.89) | 0.001 |
| Model 2 | 1.00 (reference) | 2.42 (1.94 - 3.00) | <0.001 | 0.76 (0.64 - 0.90) | 0.002 |
| Model 3 | 1.00 (reference) | 2.21 (1.76 - 2.78) | <0.001 | 0.78 (0.65 - 0.92) | 0.004 |

Abbreviations: HR, Hazard Ratio, CI, Confidence Interval.

Model 1 adjusted for age, sex (male/ female) and BMI.

Model 2 additional adjusted for ethnic (white/ other), education (college or university / other) and Townsend index.

Model 3 additional adjusted for smoke (yes / no), drinks (yes / no), physical activity (at goal / not at goal) and healthy diet.

**Table S2**. Excluding participants with osteopenia (T-score < −1) from the baseline.

| Incident osteoporosis | Cox regression [HR (95%CI), *p* - value] | | | | |
| --- | --- | --- | --- | --- | --- |
|  | Normal pace | Slow pace | | Brisk pace | |
| Model 1 | 1.00 (reference) | 2.32 (2.11 - 2.56) | <0.001 | 0.85 (0.79 - 0.91) | <0.001 |
| Model 2 | 1.00 (reference) | 2.21 (2.00 - 2.44) | <0.001 | 0.86 (0.80 - 0.92) | <0.001 |
| Model 3 | 1.00 (reference) | 2.03 (1.82 - 2.26) | <0.001 | 0.87 (0.81 - 0.94) | <0.001 |
| Incident fracture | Cox regression [HR (95%CI), *p* - value] | | | | |
|  | Normal pace | Slow pace | | Brisk pace | |
| Model 1 | 1.00 (reference) | 2.91 (2.13 - 3.97) | <0.001 | 0.79 (0.61 - 1.02) | 0.068 |
| Model 2 | 1.00 (reference) | 2.82 (2.05 - 3.87) | <0.001 | 0.80 (0.62 - 1.04) | 0.100 |
| Model 3 | 1.00 (reference) | 2.47 (1.76 - 3.46) | <0.001 | 0.81 (0.62 - 1.06) | 0.127 |

Abbreviations: HR, Hazard Ratio, CI, Confidence Interval.

Model 1 adjusted for age, sex (male/ female) and BMI.

Model 2 additional adjusted for ethnic (white/ other), education (college or university / other) and Townsend index.

Model 3 additional adjusted for smoke (yes / no), drinks (yes / no), physical activity (at goal / not at goal) and healthy diet.
